# Editorial: Applications and advances of carbon-based materials in electrochemistry

**DOI:** 10.3389/fchem.2026.1862084

**Published:** 2026-05-22

**Authors:** Min Cui, Ping Li, Yang Liu, Shuai Wang

**Affiliations:** 1 School of Chemistry and Chemical Engineering, Qilu University of Technology (Shandong Academy of Sciences), Jinan, China; 2 School of Materials Science and Engineering, Linyi University, Linyi, China; 3 School of Chemistry, Chemical Engineering and Biotechnology, Nanyang Technological University, Singapore, Singapore

**Keywords:** carbon dots, carbon-based materials, design principles, electrochemistry, environmental sensing, polymer-carbon interfaces

## Introduction

Carbon-based materials have transcended their traditional role as passive conductive supports to become active modulators of electrochemical interfaces across energy conversion, biosensing, and environmental monitoring. This Research Topic consolidates advances demonstrating how rational design of carbon architectures, from zero-dimensional quantum dots to three-dimensional printed scaffolds, unlocks performance breakthroughs by simultaneously optimizing electronic conductivity, active site accessibility, and interfacial charge transfer. The four collected articles span the electrochemical application spectrum, revealing common design principles that bridge molecular recognition, catalytic transformation, and materials processing.

## Bioelectrocatalysis: carbon nanomaterials as enzyme activators

The integration of biological recognition elements with synthetic carbon nanomaterials represents a critical frontier in personalized healthcare and food safety monitoring. Guan et al. provide a comprehensive review of glucose oxidase (GO_x_)-based electrochemical biosensors, tracing the evolution from oxygen-dependent first-generation devices to mediator-free fourth-generation systems (*Front. Chem.* 2025, *13*, 1591302). The authors identify dual innovation pathways: nanomaterial engineering through graphene oxide, carbon nanotubes, and MXenes that establish direct electron transfer (DET) channels, and enzyme chemical modification via ferrocene conjugation or hydrophobic surface functionalization that enhances thermal stability (up to 3.7-fold improvement at 80 °C). Critically, this work challenges the prevailing focus on nanomaterial optimization alone, demonstrating that synergistic enzyme-carbon interface design achieves 221.0 μA mM^-1^·cm^-2^ sensitivity with 82% activity retention after 30 days, performance metrics approaching continuous glucose monitoring requirements for diabetes management.

## Electrocatalysis: carbon dots as crystal growth modulators

Addressing the kinetic bottleneck of oxygen evolution reaction (OER) in water electrolysis, Wang et al. introduce a one-pot hydrothermal oxidation strategy that simultaneously cleaves petroleum coke into hydroxyl-functionalized carbon dots (HO-CDs) and grows Ni(OH)_2_ *in situ* on nickel foam (*Front. Chem.* 2025, *13*, 1656451). The mechanistic innovation lies in HO-CDs acting as morphological modulators rather than mere conductive additives, their presence suppresses oriented Ni(OH)_2_ crystallization, creating nanostructures with abundant edge sites while inducing electron redistribution that elevates Ni oxidation states. This electronic coupling optimizes oxygen intermediate binding energetics, yielding 353 mV overpotential at 50 mA cm^-2^ with 81.2 mV dec^−1^ Tafel slope and 92% current retention after 24 h. The work exemplifies interfacial chemistry-driven catalyst design, where *in situ* carbon dot incorporation during metal hydroxide growth generates heterostructures unattainable through post-synthesis mixing.

## Environmental sensing: additive manufacturing democratizes electrode fabrication

The transition from laboratory prototypes to field-deployable sensors demands scalable, customizable fabrication approaches. Shaw et al. demonstrate the first fully 3D-printed carbon electrode for environmental pollutant detection, formulating a carbon nanofiber-graphite-polystyrene composite ink compatible with fused deposition modeling (*Front. Chem.* 2025, *13*, 1655841). Applied to 2,4-dinitrophenol (2,4-DNP) quantification via double potential step chronoamperometry, the printed electrodes achieve 7.8 μM detection limits with 106% recovery in pond water samples. Beyond analytical performance, this work validates on-demand sensor manufacturing: electrode geometry, composite ratio, and surface activation can be iteratively optimized through digital design without cleanroom infrastructure or lithographic processing. The approach democratizes electrochemical sensor development, enabling resource-limited settings to fabricate application-specific devices for decentralized water quality monitoring.

## Materials processing: polymer-carbon interfaces in resource recovery

Bridging fundamental electrochemistry and industrial-scale extraction processes, Ma et al. investigate how polymer binders modulate the adsorption-desorption performance of aluminum-based lithium adsorbents (Li/Al-LDH) for salt lake mining (*Front. Chem.* 2025, *13*, 1628941). Through systematic comparison of polyvinyl chloride (PVC), polyvinylidene fluoride (PVDF), and sodium alginate (SA), the authors reveal that binder hydrophilicity governs Li^+^ diffusion kinetics while crosslinking density determines structural stability under thermal cycling. SA-based composites achieve optimal performance (5.84 mg/g desorption capacity at 40 °C) by forming three-dimensional hydrogel networks that maintain ion transport pathways during volume changes. This structure-performance framework extends beyond lithium extraction to carbon-supported battery electrodes and catalyst formulations where polymer matrices must reconcile ionic conductivity, mechanical robustness, and electrochemical stability.

## Convergent design principles and future horizons

### Four unifying themes emerge across these contributions

1. Interfacial Optimization Dictates Performance

Whether enzyme-nanomaterial junctions, carbon dot-metal hydroxide heterostructures, or polymer-adsorbent composites, controlled interfacial chemistry governs charge/mass transfer efficiency more profoundly than bulk material properties.

2. Multi-Scale Hierarchical Design

High-performance systems integrate molecular modifications (enzyme surface chemistry), nanoscale engineering (carbon dot-induced crystal growth), and macroscopic structuring (3D-printed architectures), demonstrating that single-length-scale optimization has reached diminishing returns.

3. *In Situ* Synthesis Unlocks Novel Architectures

Wang et al.'s one-pot approach exemplifies how simultaneous carbon dot formation and catalyst growth creates interfacial bonding unachievable through post-synthesis mixing, a strategy applicable to other metal oxide/carbon composites.

4. Translation-Focused Validation

All studies prioritize real-sample testing (human serum analogs, pond water, brine solutions), reflecting maturation from proof-of-concept demonstrations toward commercially viable technologies.

### Critical challenges requiring urgent attention include

1. While 24-h OER stability and 30-day enzyme retention represent improvements, industrial deployment demands multi-year lifespans under variable conditions.
2. Translating hydrothermal/electrochemical methods to ton-scale production with reproducible quality remains economically prohibitive.
3. The field lacks unified benchmarking protocols, particularly for 3D-printed electrodes where printing parameters critically influence electrochemistry.
4. Carbon nanomaterial synthesis energy costs, polymer binder biodegradability, and end-of-life recyclability require lifecycle assessment frameworks currently absent from literature.

The advances showcased in this Research Topic position carbon-based materials as programmable platforms where electronic structure, morphology, and surface chemistry can be co-optimized through rational design. As machine learning accelerates materials discovery, in operando characterization elucidates degradation mechanisms, and sustainable synthesis routes valorize waste carbon sources, we anticipate carbon nanomaterials will anchor next-generation technologies addressing interconnected challenges in healthcare diagnostics, renewable energy infrastructure, and environmental stewardship.

